# AFM-IR for Nanoscale Chemical Characterization in Life Sciences: Recent Developments and Future Directions

**DOI:** 10.1021/acsmeasuresciau.3c00010

**Published:** 2023-06-16

**Authors:** A. Catarina V. D. dos Santos, Nikolaus Hondl, Victoria Ramos-Garcia, Julia Kuligowski, Bernhard Lendl, Georg Ramer

**Affiliations:** † Institute of Chemical Technologies and Analytics, TU Wien, Getreidemarkt 9, 1060 Vienna, Austria; ‡ Health Research Institute La Fe, Avenida Fernando Abril Martorell 106, 46026 Valencia, Spain

**Keywords:** AFM-IR, chemical imaging, biospectroscopy, single cell imaging, mid-infrared spectroscopy

## Abstract

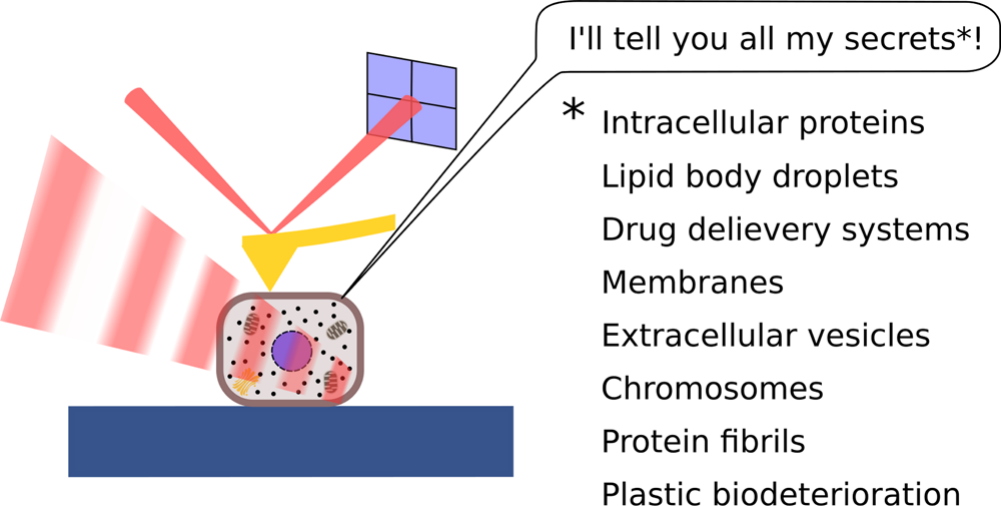

Despite the ubiquitous absorption of mid-infrared (IR) radiation by virtually all molecules that belong to the major biomolecules groups (proteins, lipids, carbohydrates, nucleic acids), the application of conventional IR microscopy to the life sciences remained somewhat limited, due to the restrictions on spatial resolution imposed by the diffraction limit (in the order of several micrometers). This issue is addressed by AFM-IR, a scanning probe-based technique that allows for chemical analysis at the nanoscale with resolutions down to 10 nm and thus has the potential to contribute to the investigation of nano and microscale biological processes. In this perspective, in addition to a concise description of the working principles and operating modes of AFM-IR, we present and evaluate the latest key applications of AFM-IR to the life sciences, summarizing what the technique has to offer to this field. Furthermore, we discuss the most relevant current limitations and point out potential future developments and areas for further application for fruitful interdisciplinary collaboration.

Atomic force microscopy-infrared spectroscopy (AFM-IR), named in some publications as photothermal-induced resonance (PTIR), is a scanning probe technique where a pulsed, tunable, IR laser is added to an AFM instrument, resulting in nanoscale IR molecular chemical information. Lateral and vertical spatial resolutions that can be achieved with AFM-IR depend on the measurement mode used (further discussed below), with tapping mode AFM-IR providing the best lateral resolution of the technique at 10 nm.^1^ This technique is still relatively recent and has been the subject of improvements in measurement speeds, types, resolution, and sensitivity since it was first published in 2005.^2^ The technical evolution of AFM-IR has been the subject of several reviews, in which the interested reader can find a detailed description of this subject.^3−5^ In this perspective, only a brief description of the main characteristics and working modes of AFM-IR will be given, with the intention of allowing readers with life sciences backgrounds to better understand the potential of this technique and its potential applications to their research.

A typical AFM-IR setup is depicted in Figure 1 in its two possible illumination geometries, bottom illumination (a) and top illumination (b), referring to the positioning of the IR laser relative to the sample and AFM cantilever. In bottom illumination, the IR radiation hits the sample from below in an attenuated total reflection (ATR) configuration which restricts sample thicknesses to <500 nm due to an otherwise loss of signal linearity.^6^ The prism is composed of an IR-transparent material, most commonly ZnSe, which may place restrictions on sample and sample preparation protocols due to its chemical incompatibility with acids. However, bottom illumination also has advantages, namely the possibility of carrying out measurements in liquid, and the use of cantilevers made from IR absorbing materials such as silicon and silicon nitride, whose use is restricted in top-illumination configurations.^7^ In top illumination geometries there is more freedom in the choice of substrate, which may include silicon wafers and gold-coated substrates, in addition to mid-IR-transparent materials (CaF_2_, ZnS, ZnSe, etc.),^8^ which opens the way for a higher variety of sample preparation protocols. The positioning of the IR laser path directly hits the cantilever in this configuration, which therefore needs to be metal-coated to prevent undesired contributions to the signal by IR absorption of the cantilever itself. When gold-coated cantilevers and substrates are used either alone or in combination, an enhancement of the AFM-IR signal up to 8-fold can be obtained;^9,10^ however, care must be taken when interpreting the resulting AFM-IR spectra due to the wavelength-dependence of this phenomenon.^11^

**Figure 1:**
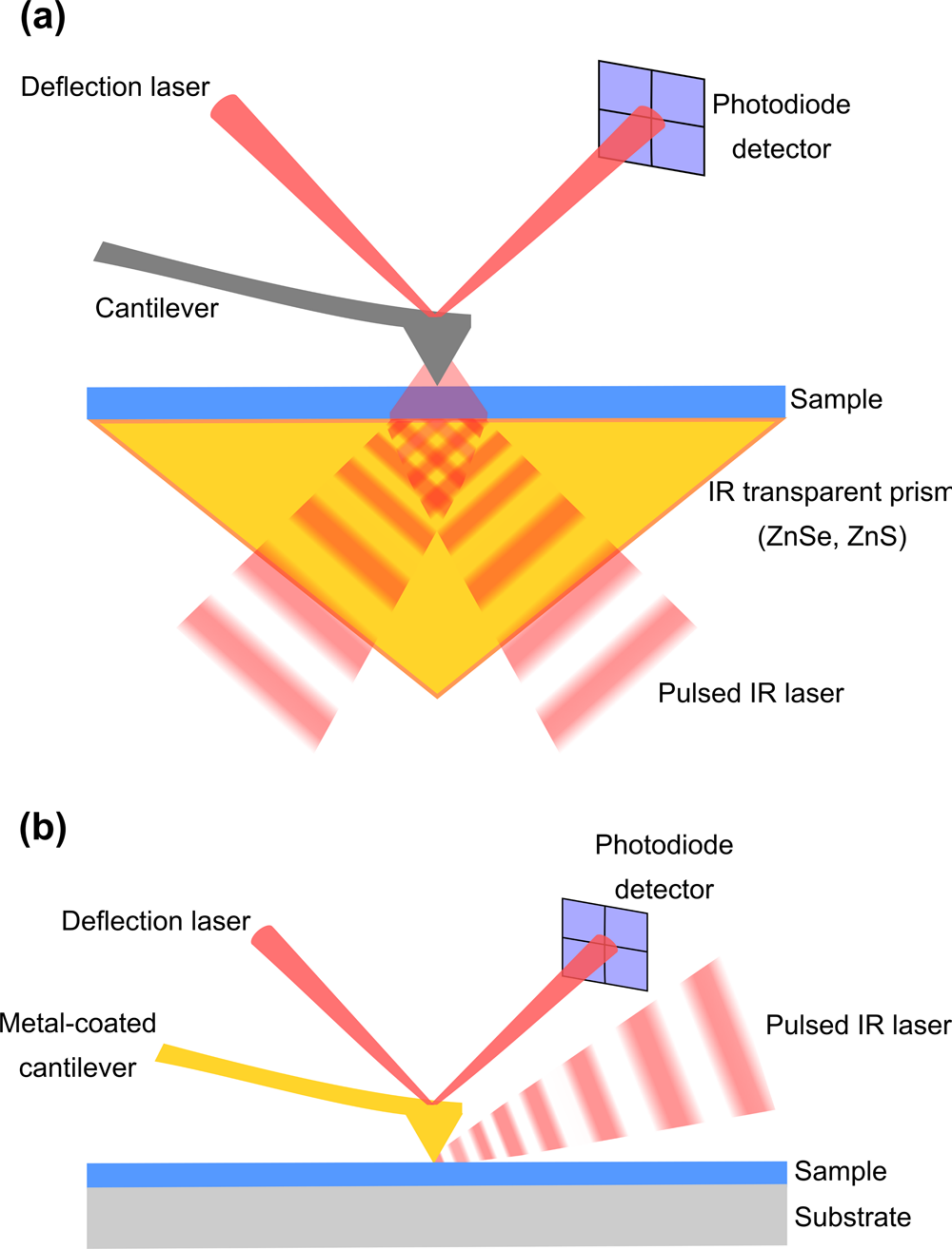
Schematic representation of the two possible AFM-IR illumination geometries: (a) bottom illumination and (b) top illumination.

The basic working principle of AFM-IR is the thermal expansion of the sample area below the AFM tip upon absorption of the IR laser radiation which is focused at this location. During the on phase of the laser cycle, the sample absorbs IR photons and the molecules convert to an excited vibrational state. When returning to the ground vibrational state some of the energy is lost in the form of heat through the lattice, which in turn is dissipated through mechanical expansion.^5,12^ This quick expansion “hits” the cantilever resulting in an oscillation motion which is picked up through the cantilever deflection signal and is proportional to the wavelength-dependent absorption coefficient, as is the case for classical IR transmission spectroscopy.^12^ This means that when interpreting an AFM-IR spectrum, one can make use of the many spectra-structure correlations that have been established over the years for Fourier-transform IR spectroscopy (FTIR), a critical advantage. AFM-IR is able to circumvent the diffraction limit and reach spatial resolutions within tens of nanometers due to the signal detection occurring only on the portion of the sample directly below the tip and not on the whole area of the sample illuminated by the IR laser spot.^9^ In AFM-IR there are two possible ways to record data, one being taking a full-length spectrum by sweeping the wavelength range of the laser used and the other collecting an absorption map at a chosen wavenumber.

It should be noted that AFM-IR is not the only technique capable of delivering vibrational spectroscopy beyond the resolution limit: O-PTIR (optical-photothermal infrared spectroscopy), TERS (tip-enhanced Raman spectroscopy), and s-SNOM (scattering scanning near-field optical microscopy)^13^ are examples of such techniques. In O-PTIR the signal is generated in a similar fashion to AFM-IR: a pulsed, tunable IR laser aimed at the sample causes thermal expansion upon radiation absorption. The main difference between these two techniques is the detection of the expansion, which is carried out using a visible laser in the case of O-PTIR, meaning that it is not a scanning probe technique and has a higher potential throughput.^14^ Furthermore, the presence of the visible laser allows for the simultaneous collection of Raman spectra, thus providing extra information.^15^ The resolution limit of O-PTIR is determined by diffraction limit of the visible laser and can reach 400–500 nm,^16^ which is lower than that of AFM-IR. TERS on the other hand, is a scanning probe technique that takes advantage of metal-coated AFM tips or etched conductive scanning tunneling microscopy (STM) wire tips to enhance the electromagnetic field in the vicinity of the tip, leading to stronger Raman signals with subnanometer resolutions and sampled depths of a few nanometers.^4^ The spatial resolution of TERS depends on the experimental conditions (air or vacuum, room temperature or lower temperatures), with typical values ranging from 3 nm to 5 nm^4^ and sub nanometer resolutions (1.5 Å) have also been reported.^17,18^ Thus, AFM-IR and TERS can be used in a complementary manner since their typical resolutions and probed depths do not overlap. For a more detailed description of TERS, as well as a review of some of its applications to biological samples, a review by Kurouski et al.^4^ is recommended. In s-SNOM, (infrared) light scattered from a metalized AFM tip is collected and analyzed.^13^ This allows access to information on the sample’s optical properties (e.g., its complex refractive index) in the near-field around the tip with lateral spatial resolutions of 5 to 170 nm, depending on the sharpness of the tip used.^19,20^ s-SNOM is not limited to the mid-IR range,^19^ but its application using mid-IR to biological samples has been recently reviewed by Wang et al.^21^

AFM-IR can be operated in three main modes, distinguished by the type of AFM mode of operation and by the way the AFM-IR signal is modulated: ring-down contact mode, resonance-enhanced contact mode, or in tapping mode. Additionally, two recently developed modes are peak-force tapping IR (PFIR)^22^ and surface sensitive AFM-IR.^23^ The first mode to be developed was the ring-down contact mode, in which upon IR absorption and thermal expansion of the sample the cantilever (which is always in contact with the sample) is struck into a slowly decaying oscillation (slow in comparison to the time scale of the thermal expansion), thus producing a ring-down pattern. In this mode, several of the cantileveŕs mechanical modes are excited, and the AFM-IR signal is obtained either from the amplitude of a specific mode or from the peak-to-peak of the cantilever’s oscillation, both of which are directly proportional to the wavelength-dependent absorption coefficient of the sample.^12^ Measurements using this mode are usually carried out with optical parametric oscillators as the pulsed IR source which have high power output and short pulse lengths (<10 ns).^24^ Ringdown measurements have lower sensitivity than resonance-enhanced contact mode measurements (see below)^23^ but yield similar spatial resolutions (lateral resolution of both modes ≈20 nm,^9,25^ probe depth of >1 μm).^3,27^

The second option when measuring in contact mode is the resonance-enhanced contact mode, in which the pulse rate of the IR laser is set to match one of the mechanical contact resonance frequencies of the cantilever, leading to an amplification of the deflection amplitude and, consequently, of the signal obtained.^23^ For typical AFM contact mode cantilevers, contact resonance frequencies lie in the range of several tens to several hundreds of kilohertz and depend on cantilever properties as well as local sample mechanical properties and measurement settings. Hence, this mode requires the use of a laser source capable of reaching and rapidly adjusting high pulse repetition rates in the range of hundreds of kilohertz, usually a quantum cascade laser (QCL). Furthermore, since the enhancement is dependent on the laser repetition rate matching the contact resonance frequency of the cantilever, some sort of resonance tracking is required to ensure that this condition is met, even when the AFM tip scans through materials with different mechanical properties. When measuring spectra this is usually achieved by quickly scanning a set range of frequencies around the original resonance position and adjusting the repetition rate accordingly (chirp) once before each spectrum;^27^ however, when recording images a phase-locked loop (PLL) is used instead, which relies on phase angle rather than amplitude to more quickly monitor and react to changes in the peak position. Resonance tracking during AFM-IR imaging is not always able to properly compensate for the shifts observed when measuring samples with large disparities in mechanical properties such as polymer blends,^28^ or soft liposomes deposited on a hard substrate. An alternative approach to this problem using a closed-loop piezo controller allowing for off-resonance measurements has also been described.^29^ When combined with gold coated cantilevers and substrates, resonance-enhanced contact mode AFM-IR can reach monolayer sensitivity due to “lightning-rod” enhancement.^9^

Tapping mode AFM-IR was first published in 2018, making it the most recent of the three main AFM-IR modes.^30^ In this mode, the cantilever is oscillated at one of its resonance frequencies (e.g., *f*_1_) and the signal detection occurs at another resonance frequency (e.g., *f*_2_) in a heterodyne detection scheme. To achieve this, the laser repetition rate *f*_L_ is set to *f*_2_ – f_1_, which usually requires the use of a QCL, as the *f*_L_ for commonly used cantilevers will usually have values within the range between 250 and 1500 kHz.^1,28^ Measurements where the tapping frequency used is the second resonance of the cantilever and signal detection occurs at the first resonance frequency are also possible and can result in a higher signal^31^ but are also more prone to signal instability. In tapping mode AFM-IR, the cantilever is only intermittently in contact with the sample, resulting in reduced tip–sample interactions, particularly lateral forces,^32^ which make this mode well-suited for the analysis of soft, easily damaged and/or loosely adhered (to the substrate) samples. However, tapping mode AFM-IR yields lower intensity signals, which may lead to the use of higher laser power that requires care not to damage the sample. An important advantage of tapping mode AFM-IR is the lower sensitivity of the resonance frequency to changes in a sample’s mechanical properties,^33^ which can be of relevance, for instance, in polymer samples.^28^ This does, however, have its limits, and in extreme cases the use of a PLL to track and adjust the laser frequency (similarly to the resonance-enhanced contact mode) is advisible.^23^ The best lateral resolution reported for this mode is ≈10 nm,^1^ and it has been reported to achieve a probe depth of 50 nm,^23^ allowing one to monitor only the top layer of the sample.

Finally, in PFIR the tip is in intermittent contact with the sample, but unlike the tapping mode, this oscillation happens at frequencies lower than the resonance frequency.^22^ During the part of the cycle where the tip is in contact with the sample, the IR laser is triggered and, similarly to the ring-down mode, the ensuing photothermal expansion is detected by the cantilever through the deflection signal in addition to information on the sample’s mechanical properties given by conventional peak force imaging.^22^ Similarly to the tapping mode, PFIR exerts lower lateral tip–sample forces, but the IR signal acquisition is closer to that of ring-down contact mode AFM-IR,^23^ albeit with higher lateral resolutions of down to 6 nm in air^34^ and 10 nm in liquid.^35^ PFIR has been applied in the context of the life sciences. We recommend to the interested reader the following studies on cell wall particles,^34^ lignocellulosic fibers,^36^ macrophage immune regulation,^37^ and protein fibrils in liquid.^35^

Even though there is a large number of available AFM-IR modes, most studies appear to use contact mode AFM-IR. We suspect that the choice between resonance-enhanced and ring-down AFM-IR is made due to available infrastructure (older AFM-IR systems used a low repetition rate laser incapable of resonant excitation) rather than due to experimental considerations. When the contact mode is not possible due to sample properties, the tapping mode AFM-IR is used. Surface sensitive AFM-IR is not yet in widespread use, but we expect that, like tapping mode AFM-IR, it will be chosen when ordinary resonance enhanced AFM-IR is insufficient. PFIR is, at the time of writing, not available commercially and therefore not used by many groups.

AFM-IR’s chemical imaging is based on the use of mid-infrared radiation (mid-IR), which is usually defined as having wavenumbers between 400 cm^–1^ and 4000 cm^–1^. Due to these long wavelengths, mid-IR is nondestructive. Spectral features in the mid-IR correspond to the excitation of molecular vibrations, and their frequency depends on both the bond strength and the mass of the atoms involved, hence, in first approximation, mid-IR bands can be assigned to specific functional groups.^38^ Each molecule has unique energy levels of its vibrational modes, which translate into a unique IR spectrum (“fingerprint”).^39^ Furthermore, intermolecular interactions such as hydrogen bonds can have an influence on the bond strengths of the molecules they are formed from, and thus, their presence is detectable through mid-IR spectroscopy.^40,41^ Mid-IR spectroscopy is of particular interest to the life sciences because it can provide information in a nondestructive, label-free manner: all of the major groups of biomolecules (proteins, nucleic acids, lipids, and polysaccharides) are active in the mid-IR region. However, this advantage can also become a disadvantage when analyzing complex biological samples in which all above-mentioned biomolecule groups are present: the IR spectrum will be a mixture of all, and small variations can be difficult to detect and disentangle from the spectrum without recurring to chemometric modeling or the use of IR-tags. Conventional mid-IR microscopy is diffraction limited and can only reach resolutions in the order of several micrometers (from 2.5 μm to up to 25 μm as the wavelength increases).^42^ The use of attenuated total reflection (ATR) configurations can improve the resolution to the range of 2–4 μm,^43^ which nonetheless hampers its widespread application, since many intracellular components have smaller dimensions, hence the usefulness of a technique like AFM-IR. It is however important to highlight that the spectral range of AFM-IR is in practice limited by the type of laser used, potentially meaning that not all of the mid-IR spectral features of a target analyte will be visible in the AFM-IR spectra using a specific light source. For example, the widely used multichip QCL systems that are currently commercially available typically cover ≈1000 cm^–1^ by combining several individual external cavity-QCLs in one housing. In the following paragraphs, a brief overview over the most prominent mid-IR spectroscopic features of each biomolecule group is provided (Figure 2), for deeper insights into the applications of conventional mid-IR spectroscopy methods to the life sciences, the following reviews are suggested.^44−48^

**Figure 2:**
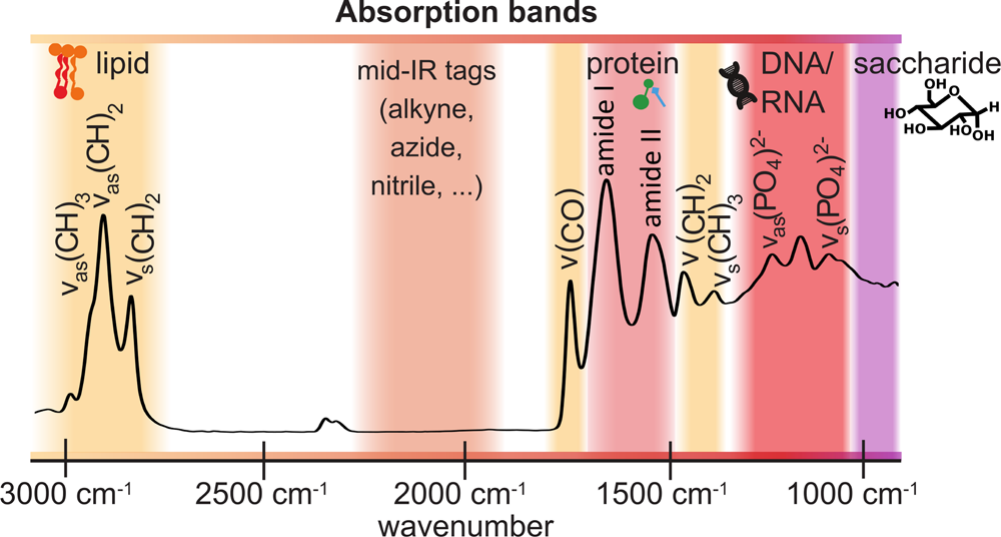
Absorption bands of biomolecules and mid-IR tags in the mid-infrared region.

Proteins have five dominant bands in the mid-IR spectra: the amide I (1600 cm^–1^ to 1700 cm^–1^, usually centered around 1650 cm^–1^), amide II (centered around 1550 cm^–1^), amide III (from 1400 cm^–1^ to 1200 cm^–1^), amide A (centered around 3500 cm^–1^), and amide B (centered around 3100 cm^–1^). The amide I band, caused mainly by the stretching vibration of the C=O group, is especially useful for its well-established sensitivity to a protein’s secondary structure, of which a thorough description can be found in the review by Barth.^49^ In short, β-sheet-containing proteins absorb predominantly at 1633 cm^–1^ and 1684 cm^–1^, whereas α-helix structures result in absorption around 1654 cm^–1^.^49^ In intermolecular β-sheets, the major component of amyloid aggregations produces a distinct absorption band at 1620 cm^–1^, thus allowing for the detection and characterization of protein aggregation states such as amyloid fibrils.^50^

Mid-IR spectra of lipids are generally composed of bands from the hydrocarbon chain (−CH_2_ groups) and from the polar head groups (phosphate, PO_2_^–^, and ester groups). From the hydrocarbon chain there are two groups of bands: the stretching vibrations in the region of 2950 cm^–1^ to 2800 cm^–1^ and bending vibrations at ≈1460 cm^–1^.^51^ CH_3_ groups produce bands at ≈2955 cm^–1^ and 1377 cm^–1^ and alkenes at 3010 cm^–1^ and ≈1660 cm^–1^.^51,52^ Analysis of these bands, particularly of those in the higher wavenumber region (from 2850 cm^–1^ to 3050 cm^–1^) provides information on chain length, chain unsaturation, phase transitions, and lipid orientation (when using polarized light).^51^ Furthermore, membrane phospholipids have a strong absorption band at ≈1735 cm^–1^ corresponding to the C=O vibration of ester groups, as well as bands at 1240 cm^–1^ and 1090 cm^–1^ from the phosphate groups present in the polar head.^51^

Nucleic acids, similarly to lipids, absorb at 1240 cm^–1^ and ≈1085 cm^–1^ due to the presence of phosphate groups in the backbone of both DNA and RNA.^53^ Additionally, nucleic acids also absorb around 1700 cm^–1^ to 1717 cm^–1^ (due to C=O stretching vibrations)^54^ and in the spectral range between 1520 cm^–1^ and 1663 cm^–1^ in characteristic patterns that can be attributed to specific bases and base pairings.^55^ This spectral area partially overlaps with the amide I and II bands of proteins and thus requires a careful interpretation.

Saccharides have numerous absorption bands in the mid-IR region: −OH groups absorb from 3000 cm^–1^ to 3600 cm^–1^ (stretching vibration), and CH and CH_2_ absorb both at 2800–2950 cm^–1^ and at ≈1460 cm^–1^ (as described in the lipids paragraph).^56^ However, it is the “fingerprint” region between 800 cm^–1^ and 1200 cm^–1^ that offers information on a polysaccharide’s glycosylic linkages (between 1140 cm^–1^ and 1175 cm^–1^ corresponding to a C–O–C stretching vibration) and their conformation (between 920 cm^–1^ and 1000 cm^–1^ region),^57^ as well as on the conformation of the anomeric carbon (between 800 cm^–1^ and 900 cm^–1^ region).^56,58^

Thus, combining the described wealth of chemical information provided by AFM-IR with its ability for subcellular spatial resolution at ambient conditions, it is easy to see why there has been a great interest in applying AFM-IR in the life sciences. In fact, first AFM-IR images of cells were published shortly after the technique’s invention.^2^ Due to the constant improvement and development of novel measurement modes, the types of samples that this technique can be applied to have increased tremendously in recent years, thus, without reduction of generality we focus here on the most relevant recent works (published less than five years ago) on AFM-IR in the life sciences and highlight potential directions for future research. Furthermore, we highlight key areas of future improvements, such as liquid measurements, surface sensitive mode, labeling of target molecules, and the current status of image and data treatment in the AFM-IR field and discuss advantages and applications that these can bring.

## Recent Applications to the Life Sciences

### Analysis of Whole Cells

**Figure 3:**
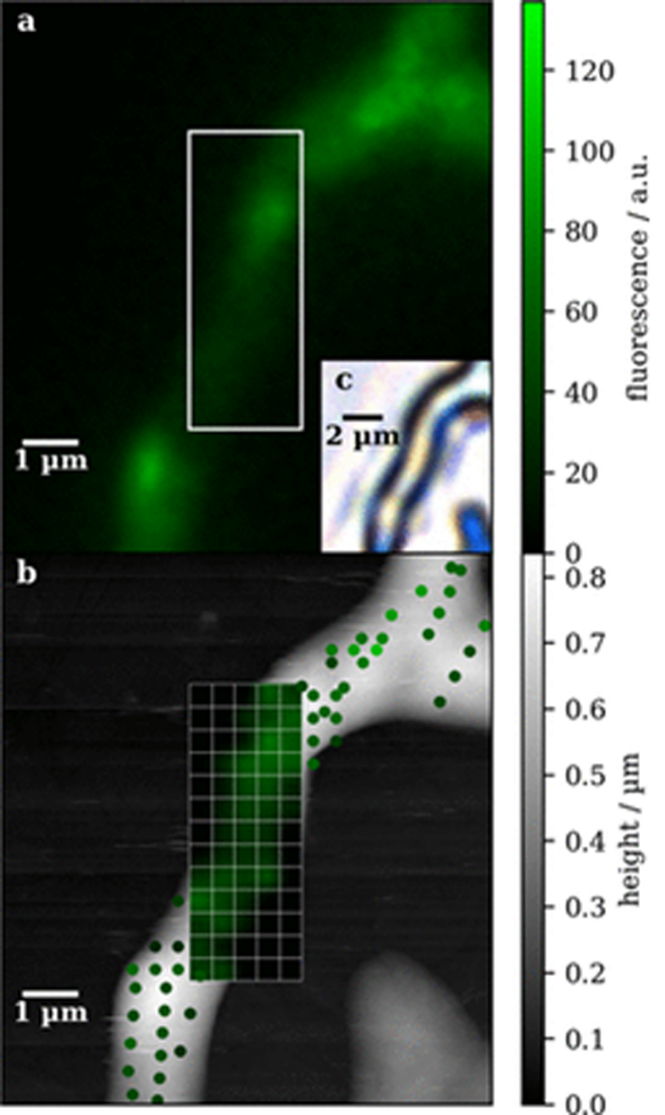
Fluorescence image (a), topography map (b), and brightfield image (c) of a *T. Reesei* hypha. Fluorescence values calculated from AFM-IR spectra [points and grid in panel (c)] using a PLS model calibrated to the presence of β-sheet-containing proteins can be compared with those measured using the fluorescence microscope (a). Reproduced from ref (62). Copyright 2020 American Chemical Society.

The analysis of whole cells using AFM-IR with the goal of identifying the intracellular distribution of its components often runs into the aforementioned blessing and curse of “everything” being IR active. With some exceptions in which the target analytes have particular IR signatures,^59^ most studies rely to some extent on the use of chemometric models to circumvent this and extract relevant information from the AFM-IR data.^60−62^ In 2020, our group published an approach to this problem which combined fluorescence microscopy and supervised machine learning with resonance-enhanced contact mode AFM-IR, with the goal of mapping the distribution of the major cellulases and xylanases within *T. Reesei*.^62^ This fungus is notorious for its well-developed enzyme secretion system essential to its survival in the wild as a saprobe^63^ and which has been leveraged in numerous industrial processes including recombinant protein production.^64^ The strain used in this study, QM6a SecEYFP, has been modified to express yellow fluorescent protein (EYFP) under the same conditions of the main cellobiohydrolase, CBHI, and to follow the same intracellular path. Therefore, in this strain the EYFP and its respective fluorescence signal are colocalized with the cellulases and xylanases, allowing for a correlation between fluorescence intensity and the presence of proteins containing β-sheet secondary structures in the same location. This was the basis for the training of a partial least-squares (PLS) model to predict cellobiohydrolase abundance from AFM-IR spectra. This model could achieve a root mean squared error (RMSE) of 13% when applied to a data set which had not been used for model training. Interestingly, the selectivity ratio (SR) showed the highest contributions to the model coming from the amide I band wavenumbers related to β-sheet secondary structures and CH_2_ stretching vibrations (lipids). This is in line with the main secondary structure motifs of several of the most abundant cellulases and xylanases, as well as with the expected intracellular path of these proteins leading up to secretion in which they are enveloped in the lipid bilayers of several organelles and later inside vesicles. Thus, it was possible to obtain a PLS model capable of mapping the intracellular distribution of the main cellulases and xylanases in *T. Reesei* (Figure 3).

Another approach was carried out by Liu et al. in 2021 for the analysis and comparison of protein expression in macrophages using AFM-IR (resonance-enhanced contact mode for single-point spectra and tapping mode for imaging) and a chemometric model to identify the subcellular differences between two *a priori* known groups of macrophages.^61^ Depending on the stimulus present in an environment, macrophages express different phenotypes, allowing them to adapt and take over different functions as necessary. These phenotypes can be divided into two broad categories: M1 macrophages that participate in pro-inflammatory responses, and M2 macrophages which participate in anti-inflammatory responses. In this study, macrophages were polarized *in vitro* and their protein expression at the subcellular scale was studied using resonance-enhanced contact mode AFM-IR for single spectra and tapping mode AFM-IR for chemical imaging. After collecting spectra at several locations in the central portions of macrophages belonging to group M1, M2, and M0 (nondifferentiated control group), the data was analyzed using principal component analysis (PCA). The three different macrophage groups clustered separately, with major differences between groups appearing in the amide I spectral region. M1 macrophages had β-sheet as the most common secondary structure motif (35% antiparallel and 5.6% parallel), whereas in M2, α-helix was the most common (38.8%). The different secondary structure detected by the AFM-IR spectra is in agreement with previous studies on protein expression of polarized macrophages. Chemical images obtained at selected amide I wavenumbers showed different patterns of protein distribution in the two groups, with the highlight being the detection of antiparallel β-sheet rich nodes in the extremities of M1 macrophages corresponding to TNF-α, a pro-inflammatory factor.

An example of whole cell imaging without resorting to chemometric models to detect the target analyte is the study by Deniset-Besseau et al.^65^ of lipid body droplet formation in *P. kesslerii*, a microalgae used in biofuel production. This organism produces large quantities of triacylglycerol (TAG)-containing lipid bodies, which due to their unique spectral signature can be located and imaged without the use of labels or chemometric approaches. Furthermore, a correlative approach was used combining ring-down contact mode AFM-IR in bottom illumination with fluorescence microscopy, which permitted the mapping of TAG lipid droplets (through AFM-IR) and their relationship to chloroplast location (mapped with fluorescence microscopy). As the cells begin producing lipid droplets, these accumulate in the center of the cell, pushing the chloroplasts to the edges.

**Figure 4:**
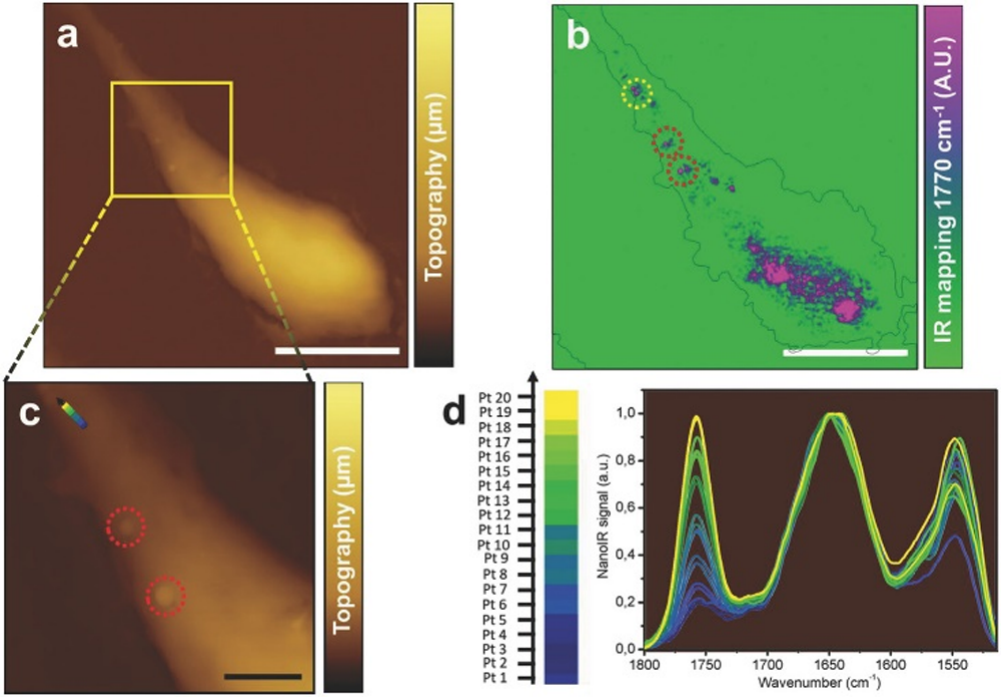
Label-free AFM-IR imaging of NPs inside a macrophage. (a) and (c) AFM topography maps with some polymeric NPs visible in panel (c). (b) AFM-IR absorption map taken at 1770 cm^–1^, a marker band for the NPs. (d) AFM-IR point spectra taken in the points marked in panel (b). Reproduced with permission under a Creative Commons Attribution 4.0 International License from ref (66). Copyright 2018 Wiley-VCH.

Another study by Pancani et al.^66^ focused on the imaging with nanoscale resolution of the location of polymeric nanoparticles typically used for drug-delivery inside macrophages (Figure 4). This study was conducted using resonance-enhanced contact mode AFM-IR with no additional labeling of the nanoparticles (NPs), taking advantage of the fact that the main absorption peak of the NPs studied (1730 cm^–1^) does not overlap with major absorption peaks of the cells analyzed. When these conditions are met, AFM-IR has a critical advantage over other high-resolution imaging methods such as fluorescence microscopy techniques, which always require the presence of a fluorophore to label the analyte of interest.

Beyond the studies summarized here, AFM-IR has also been applied to red blood cells (including pathological states)^60,67−69^ and cancer cells,^70−72^ as well as on isolated viruses.^73,74^

We would like to emphasize that whole cell analysis using AFM-IR would benefit significantly from more robust liquid measurement AFM-IR mode. Current work on liquid AFM-IR does not change the medium composition or enable long-term measurements of live cells. Thus, microfluidic integration, which allows for the monitoring of cells in a controlled environment and the ability to add nutrients and reagents in a controlled way would enable a wealth of new studies on cell response to stimuli. Furthermore, whole cell AFM-IR analysis yields complex data sets that often require resorting to multivariate models. The lack of community-accepted best practices with regard to multivariate evaluation makes it hard to compare different studies, and the lack of community-accepted software packages for data processing is a barrier for newcomers.

### Analysis of Tissues

**Figure 5:**
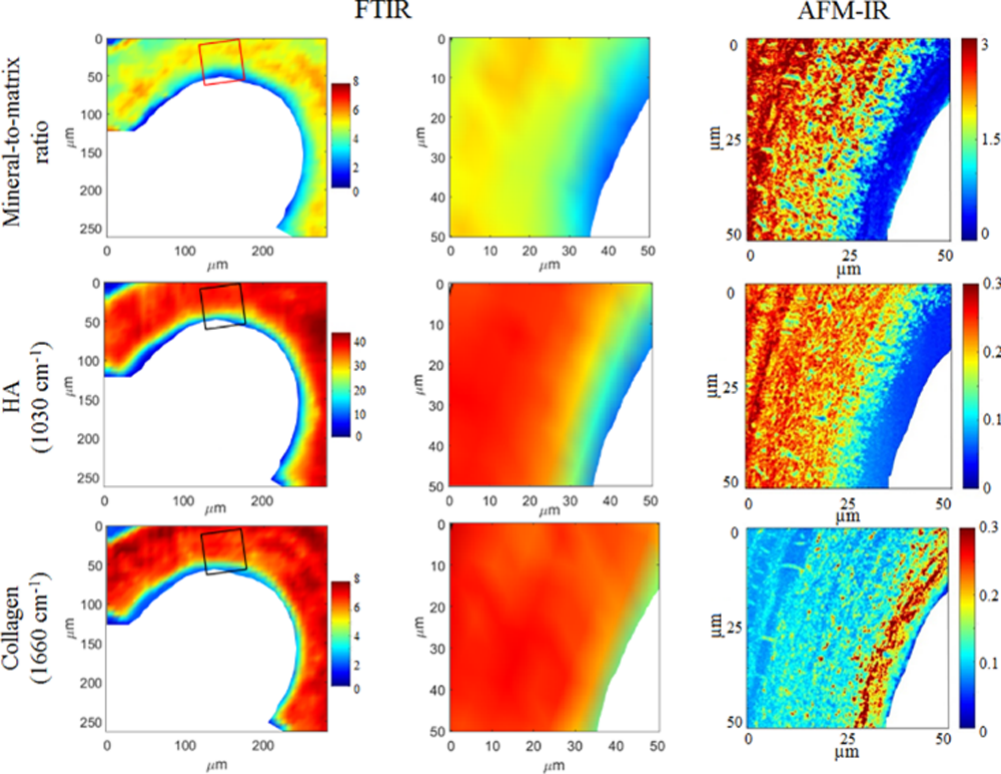
FTIR (left and middle column) and AFM-IR absorption ratios of sheep cancellous bone (right column). Here the superior resolution of AFM-IR allows for a much more detailed analysis of the distribution of the mineral-to-matrix ratio (first row), hydroxyapatite content, HA (second row), and collagen distribution (third row). AFM-IR enables the identification of a collagen-rich, nonmineralized area (osteoid). Reproduced with permission under a Creative Commons Attribution 4.0 International License from ref (75). Copyright 2018 Public Library of Science.

In the histological context, AFM-IR has been employed in the characterization of different tissues, providing chemical information at the nanoscale complementary to other traditional tissue analysis methods. Bone tissue has been studied by Imbert et al.^75^ using ring-down contact mode AFM-IR, and by Ahn et al.^76^ using both ring-down and resonance-enhanced contact mode. Despite using bone tissue from two different mammals (sheep and mouse), both studies observed an increase in mineralization with bone tissue age and were able to map this distribution using AFM-IR (Figure 5). Furthermore, an alternating pattern with 2–8 μm periodicity consisting of higher and lower mineral-to-matrix ratios was identified as being the lamellae, a key structural element in mammalian bone.^75^ Qian et al.^77^ performed a study comparing the nanoscale properties of bones affected by osteoporosis with healthy controls using resonance-enhanced contact mode AFM-IR. Bone sections from osteoporosis patients were found to have a less mature mineral matrix than that of the control group, which can be associated with reduced stability.^77^ Further related studies include AFM-IR analysis of bone-like nodules formed by stem cells,^78^ of bone composite xenografts,^79^ and of human dentin.^80^

We would also like to highlight recent studies focusing on characterizing human hair samples at the nanoscale using AFM-IR.^81−84^ This includes the hair medulla,^82^ cuticle,^81^ and cortex.^83^ Other tissues analyzed include the anterior cruciate ligament,^85^ stratum corneum^86^ (as well as drug penetration on the skin),^87^ and breast cancer,^88^ which further expand the application of this technique to the histological context.

### Biodeterioration Studies

AFM-IR has been applied to study the biodeterioration of polyurethane films by the yeast *P. laurentii* in a 2020 study by Barlow et al.^89^ The yeast was incubated for 30 days on two ZnSe substrates spin coated with two different types of polyurethane films and then measured using a ring-down contact mode in a bottom-illumination configuration. The yeast cells formed monolayer aggregates over the urethane and deteriorated the polymer through both ester hydrolysis and bulk loss. The authors had to deal with cell translocation and microplastic erosion due to the incubation conditions of up to 95% relative humidity and further influenced by biofilm production, which caused water condensation on the sample surface. Through the acquisition of chemical image ratios, the authors showed that this biodegradation is most pronounced at the edges of cells or cell clusters and results in a depression in the urethane coating where extracellular polymeric substance (EPS) residues typical of biofilms are left. Several components of EPS could be detected in the spectra, including polysaccharide and protein residues in addition to urethane-rich secondary microplastics. The edge degradation pattern observed in the study is attributed to the high affinity between the hydrolases secreted by *P. laurentii* and the polymer substrate (particularly the ester moieties) which “traps” them in the polymer directly near the cell. The degradation of the coated film and subsequent viability tests show that at least some of the cells were able to remain alive and degrade the coating for a time. However, in the absence of nitrogen sources other than dead cells, most cells eventually died or became metabolically inactive. This raises the question of whether longer term observations of deterioration processes might occur under more favorable conditions for cellular growth, e.g., in the presence of liquid growth medium. The cells tested for viability in this study were, however, not the same cells that underwent AFM-IR measurements and were placed on glass and not ZnSe substrates.^89^ It would be important to establish whether it is possible for cells to survive (a) on commonly used AFM-IR substrates and (b) AFM-IR measurements, and under which conditions (laser power, air vs liquid medium, and under which ambient conditions). A 2018 study by Kochan et al.^90^ claims that the majority of the bacteria present on the substrate survives AFM-IR measurement conditions; however, it is not clear that the single bacteria subject to AFM-IR measurements did, and thus, this remains an open question.

### Lipid Membrane Studies

AFM is a common technique for the study of biological lipid membranes as it provides high-resolution imaging of the different components, of lipid phase separation, and of the organization of membrane proteins.^91^ Using AFM-IR, it is possible to combine this information with infrared spectroscopy, allowing for the chemical identification of the membrane components in addition to their spatial distribution. This has been demonstrated in a 2019 study by Lipiec et al., which studied the distribution of components in an artificial membrane system composed of two lipids, sphingomyelin (SM) and cholesterol (Chol), and one peptide, cyclosporin A (CsA), using resonance-enhanced contact mode AFM-IR.^52^ First, by acquiring spectra using the *p* and *s* polarizations of the infrared laser, the authors were able to detect the orientation of some functional groups. The hydrocarbon chains (CH_2_) orient perpendicular to the surface of the substrate, whereas the amide and unsaturated groups in SM appear to be oriented parallel to the substrate. The studied membrane system phase separates into circular domains containing higher surface densities of SM surrounded by a Chol phase. As the percentage of Chol in the system increased, the peptide component, CsA, moves from a homogeneous distribution to a preferential location in the Chol domains where it forms complexes with SM and Chol molecules akin to those present in lipid rafts.

The study by Lipiec et al.^52^ opens the way for more biomembrane studies using AFM-IR, perhaps focusing on membrane proteins and making used of the several established protocols for membrane sample preparation used in AFM.^92^ Indeed there is literature published on light-induced conformational changes of membrane proteins in purple membranes detected using resonance-enhanced contact mode AFM-IR.^93,94^ Although purple membranes are known for being particularly good subjects for AFM analysis due to their 2D crystal structure,^95^ these results are nonetheless encouraging and AFM-IR analysis of biomembranes is a promising field.

### Extracellular Vesicle Characterization

Extracellular vesicles (EVs) are membrane-bound vesicles secreted by both prokaryotic and eukaryotic cells that can transport proteins, lipids, as well as nucleic acids, thus serving as important intercellular messengers.^96−98^ Exosomes are EVs with sizes from 20 to 150 nm diameter and are commonly found in bodily fluids such as milk, tears, and blood.^98^ Larger EVs with diameters between 100 nm and 1 μm are classified as microvesicles.^98^ EVs can trigger a number of responses in the target cells including apoptosis, immune-responses (by acting as antigen-presenting vesicles), and overall tissue regeneration.^98^ Furthermore, under pathological conditions EVs also play a role, e.g., in the spread of cancer metastases and in neurodegenerative disorders through the spread of toxic protein aggregates.^98^ Due to their minute size, the analysis of individual EVs is challenging and inaccessible to most conventional methods which focus instead on bulk measurements or provide no direct label-free chemical information on individual EVs.^96,100,101^

**Figure 6:**
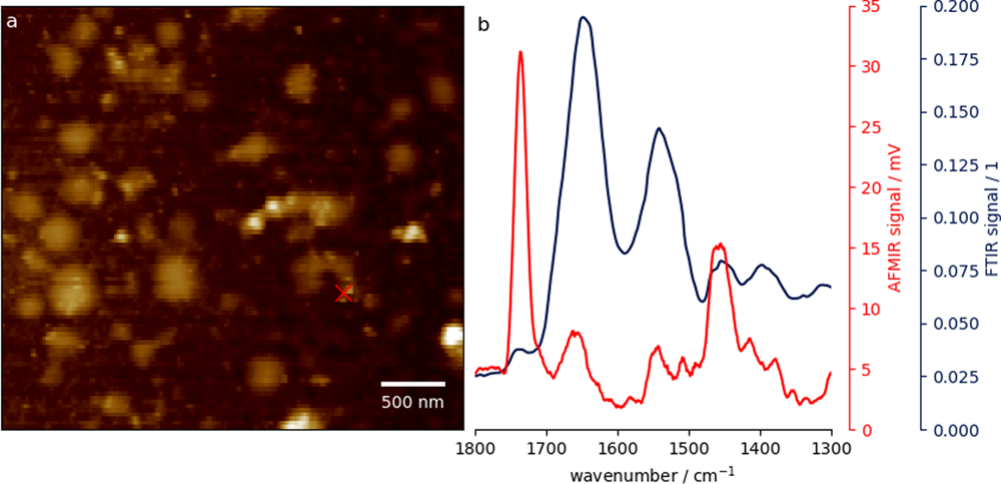
Baseline-corrected tapping mode AFM topography map (a) and AFM-IR (in red) and bulk FTIR (in blue) spectra (b) of a human milk EVs showing protein and lipid spectral signatures. EVs were immobilized on the surface of a silicon wafer using microcontact printed anti-CD9 antibodies and measured using tapping mode AFM-IR with *f*_2_ = 1500 kHz, *f*_laser_ = ≈1250 kHz, and detection at *f*_1_ = 250 kHz (measured on a Bruker nano-IR 3s; IR laser source: MIRcat-QT from Daylight Solutions; cantilever: Tap300GB-G from BudgetSensors; laser power: 49.6%; laser duty cycle: 16%). Spectrum b is an average of three spectra taken at the same location [marked with a cross in panel (a)] and smoothed using a Savitzky-Golay filter (20 points, second order).^99^

In 2018, Kim et al.^97^ reported the first analysis of EVs with AFM-IR using a bottom illumination configuration and contact mode ring-down measurements. In this study, the authors were able to obtain AFM-IR spectra of isolated EVs and their content, identifying the presence of proteins and nucleic acids and the variations of these between two EV populations and also within different EVs of the same population.^97^ The sample preparation in this study was simple, consisting of depositing a droplet of water containing isolated EVs on a ZnSe prism. Depositing particles on a substrate as done in this study can lead to the accumulation of particles on a ring-shape once the liquid evaporates called the coffee ring effect. This can be detrimental to the analysis by limiting the number of EVs that can effectively be individually studied, as particles in the ring tend to overlap. To tackle this problem, our group has recently developed a protocol that allows for the immobilization of EVs onto a silicone surface using microcontact printed anti-CD9 antibodies (Figure 6). In comparison to Kim et al.,^97^ where the EVs are placed and dried on a zinc selenide prism, here they are placed on a functionalized silicone surface, a more chemically resistant and versatile substrate. By using the anti-CD9 antibodies, the coffee ring effect can be prevented, which permits the access to more EVs. Furthermore, as only EVs are immobilized by anti-CD9, the selectivity is increased significantly, as residues from the purification steps can be washed away before tapping mode AFM-IR measurements are performed. This protocol has been applied with promising results to human milk EVs (Figure 6), which play an important role in early life immunity and intestinal development.^96^ AFM-IR spectra obtained from immobilized EVs display marker bands (Figure 6, b) in agreement with those obtained from bulk FTIR studies,^102^ thus showing the suitability of this approach for the label-free tapping mode AFM-IR analysis of human milk EVs. As demonstrated in these two approaches, AFM-IR can be applied to the nanoscale label-free chemical analysis of individual EVs and thus provide a valuable contribution to EV research.

### Chromosome and DNA Studies

AFM-IR can also give valuable information about DNA methylation as well as anticancer binding locations in chromosomes with characteristically high spatial resolution.^53^ Chromosomes contain two types of chromatin (i.e., DNA–protein complex), euchromatin and heterochromatin, distinguishable by the methylation of the cytosine bases and how packed the structure is. Heterochromatin is characterized by denser chromatin structures containing methylated cytosine bases, which in turn means that it is not active for transcription. Euchromatin, on the other hand, is loosely packed, and its genes are available for transcription. Using ring-down contact mode AFM-IR, Lipiec et al.^53^ mapped DNA methylation in metaphase chromosomes, arriving at the characteristic banding pattern known from immunofluorescence techniques but without the use of labeling agents. Furthermore, by applying PCA to the different areas of the AFM-IR spectra collected, the authors were able to confirm the distinction between heterochromatin and euchromatin areas. The study goes on to demonstrate the capability of AFM-IR to distinguish between active and inactive chromosomes, as well as the preferential binding site of a platinum anticancer drug (the inactive heterochromatin) through the use of a PCA to analyze spectral data. Despite the experimental complexity of this study,^53^ the results are very promising, particularly the ability to identify drug-binding sites in chromosomes, which can provide important mechanistic information on how anticancer drugs affect cells without the use of staining agents.

**Figure 7:**
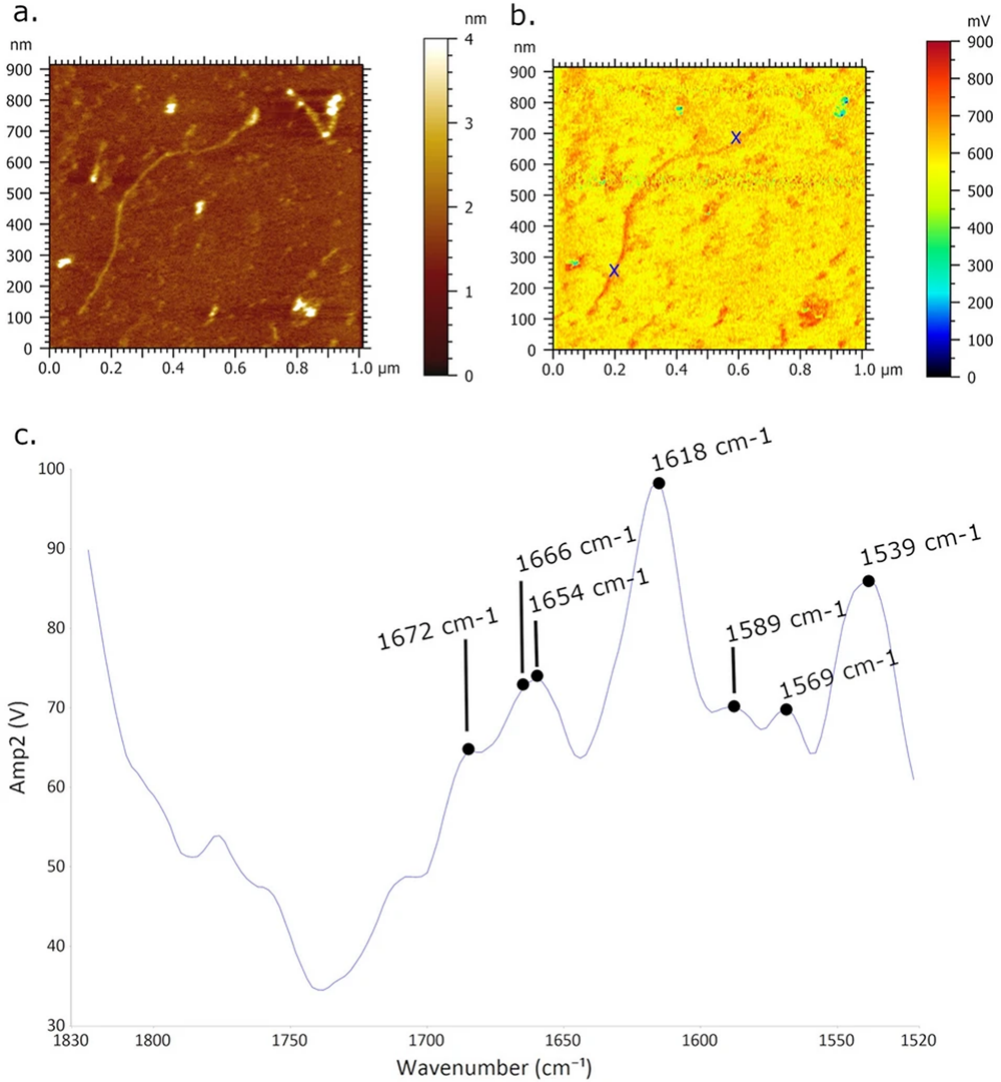
Topography map (a) and AFM-IR absorption map at 1633 cm^–1^ (b) of single DNA molecules deposited on a Ni^+^-functionalized mica surface. Average AFM-IR spectrum (c) collected at the locations marked in panel (b) show the characteristic mid-IR absorption bands of nucleic acid bases. Reproduced with permission under a Creative Commons Attribution 4.0 International License from ref (55). Copyright 2022 Nature Portfolio.

Recently, Custovic et al.^55^ published a methodological approach to analyze DNA networks and single molecules using resonance-enhanced contact mode AFM-IR. Leveraging previously available methodological approaches for DNA deposition on mica surfaces, the authors demonstrated the applicability of these methods to the AFM-IR technique and were able to acquire AFM-IR spectra and maps of DNA networks and single molecules (Figure 7). This study is an important first step in what can be the future application of AFM-IR to study DNA molecules and DNA–protein complexes.

### Studies of Protein Fibrils and Aggregates

Protein fibrils are involved in numerous neurodegenerative diseases in humans^103^ and are thus an important target of studies aiming at a better understanding of their structure, properties, and aggregation mechanisms. AFM-IR is a promising technique in this field, since it can provide information on a protein’s secondary structure at the nanoscale in a label-free manner. Rizevsky and Kurouski^104^ studied the structural features of insulin fibril aggregates using contact mode AFM-IR without recourse to labels and identified two structural polymorphs: one composed of a β-sheet core and an outer, unordered, α-helix-containing surrounding layer and another polymorph composed of a mixed β-sheet and α-helix/unordered core surrounded by an outer β-sheet structure layer.^104^ The secondary structure of the outermost layer of the fibril’s surface varies between the two polymorphs, presumably due to distinct lateral growth mechanisms.^104^ Further AFM-IR studies of protein aggregation state’s structure have been conducted on Aβ42 (present in Alzheimer’s disease),^105−108^ cell-penetrating peptides,^109^ functional amyloids,^110^ and α-synuclein (present in Parkinson’s disease),^111^ whereas other studies have focused on the effect of lipids on protein aggregation^112−114^ or the formation of amyloid microcrystals.^115^

A further step in AFM-IR studies of protein aggregation states is to investigate the interaction of these with potential drugs and drug candidate molecules. Ruggeri et al.^116^ studied the interaction between different Aβ42 aggregation species and bexarotene, a drug which delays protein aggregation. The study mainly used AFM-IR and combined it with chemometric models to allow for more effective data interpretation. This study made use of the lighting-rod signal enhancement by using gold-coated substrates and tips and was measured using short laser pulses and off-resonance to minimize sample damage and limit the contribution of the substrate to the signal obtained, which otherwise hinders the measurement of objects smaller than the diameter of the tip.^116,117^ The authors were able to distinguish between the spectral signatures of oligomers (composed of proteins in random coil conformation as well as in antiparallel intermolecular β-sheet conformation) and fibrils (composed overwhelmingly of parallel intermolecular β-sheet structures).^116^ However, the most interesting result was the observation that bexarotene molecules form hydrogen bonds linking their carboxyl groups to the Aβ42 aggregates which hinders the progression to further aggregation states such as fibrils. This study showcases the potential of AFM-IR to contribute to a better understanding of drug-target interactions.

When analyzing amyloid fibrils using AFM-IR particular care is required when the obtained spectra will be greatly affected by the experimental setup used.^118^ For highly oriented samples with less than 10 nm thickness (as can be the case when measuring amyloid fibers and other types of protein aggregation states, depending on sample preparation), a recent study by Waeytens et al. recommends using bottom illimitation, Si cantilevers, and s-polarized light to obtain closer results to those expected from classical ATR-FTIR spectroscopy.^118^ These are important considerations when setting up a study, particularly as the use of gold tips is frequent due to the signal enhancement they provide. This enhancement, however, comes at the expense of spectral reliability for structural analysis.^118^

## Future Directions and Promising New Approaches

### Liquid Measurements

The AFM-IR study of biological samples in water, their native environment, is a technique that has so far remained elusive and seldomly used.^7,119,120^ AFM-IR measurements in liquid are particularly challenging due to the extra cantilever oscillation dampening leading to lower signal-to-noise ratios, as well as the intense background absorption of water.^7^ To tackle the background absorption challenge, deuterated water can be used instead of water and measurements are carried out in bottom illumination to limit the exposure of the medium to the IR laser beam, and thus its contribution to the signal.^3^ Furthermore, the use of bottom illumination schemes requiring IR-transparent prisms typically restricts the list of potential substrates to materials such as ZnSe and CaF_2_, which are chemically incompatible with a variety of sampling protocols, e.g., those requiring the use of acidic substances, are harder to manipulate due to their brittleness, and are costly. All of these factors have contributed to the small number of recent studies employing this methodology: excluding studies carried out using peak-force infrared microscopy, an emerging subtype of AFM-IR, only two papers have been published in the past five years using AFM-IR in liquid mode.^7,23^

Nonetheless, the perspective of measurements in liquid is attractive, provided technical improvements to the technique are undertaken. These might include the development of cheaper and more easily manipulable substrates, improvements in the instrument’s measurement stability, and a better understanding of the behavior of cantilevers during an AFM-IR measurement in liquid. Recent results show that measuring in liquid using the tapping mode might be the key to solvent signal suppression, since tapping mode measurements are inherently insensitive to the acoustic waves that result from the absorption of IR radiation by the surrounding medium (water).^23^ Future interesting avenues of research for liquid AFM-IR studies include polymer biodeterioration focusing on the longer-term survival of the cell in liquid media, protein aggregation processes and how they are affected by medium components in real-time, as well as the imaging of biological processes, to name a few. A further step could be the integration of microfluidic setups with AFM-IR, as is already the case for AFM.^121^ Such addition would enable the AFM-IR of individual cells while they are exposed to different nutrients or media components or to detect the capture and analyze the properties of vesicles captured by membrane proteins.

### Surface Sensitive Mode

Recently a “surface sensitive” mode has been developed, which is a type of contact mode leveraging a heterodyne detection scheme that allows for the probing of only the upper 10–30 nm of the sample.^23^ In this mode, the cantilever is oscillated at a high frequency, *f*_1_, and the laser set to a high off-resonance frequency, *f*_laser_, such that the sum or difference of these two frequencies coincides with one of the cantilever contact resonances (ideally with a high *Q* factor).^23^ Through nonlinear interactions, the AFM-IR signal could be detected at the resonance frequency and corresponds only to the uppermost layers of the sample.^23^ Despite not requiring hardware changes to instruments already capable of tapping mode experiments, this mode is so recent (less than two years old) that, to date, there are no published studies on its application to biological samples. This mode could allow for studies targeting cell membranes (4–5 nm thick)^122^ and immediate surroundings such as cell walls without the need to isolate them from the rest of the cell. Indeed, by combining surface sensitive measurements with previously existing modes that have no probing depth sensitivity, the deeper parts of the cell can be probed in the same experiment.

### Labeling of Target Molecules

Although one of the strong suits of AFM-IR is the possibility of high-resolution label-free studies, this is not feasible in all cases, particularly when the target analytes are not chemically distinct from their surrounding environment. A glimpse into such a possibility is offered in the work by Clède et al.,^123^ in which a rhenium triscarbonyl complex (mid-IR tag) coupled to a luminescent probe allowed for the multimodal imaging of the intracellular distribution of an estrogen derivative within breast cancer cells. The multimodal probe was linked to the target molecule via “click” chemistry. More recently, the same class of rhenium tags was used to show the intracellular targets of ferrocifens, which are potential anticancer drugs.^124^ Rhenium tricarbonyls absorb strongly at around 1950 cm^–1^, an area in which biological samples tend to have little to no absorption.^123^ Besides rhenium carbonyl tags, alkynes are another class of compounds that have an application potential as mid-IR tags in AFM-IR studies. Alkynes are widely used as Raman tags due to their absorption band in the region of 2100 cm^–1^ to 2200 cm^–1^, isolated from typical biomolecule bands (Figure 2).^125^ Tagging target analytes with alkyne-containing molecules represents another potential field of investigation, albeit with one possible downside: the QCLs commonly used in AFM-IR setups often do not cover this “remote” area of the mid-IR spectrum.

Finally, isotope labeling of compounds to study processes within the cell, or even interactions between cells, is another potential avenue of study for AFM-IR. This kind of approach has been demonstrated on bacteria using O-PTIR.^126^ The similar signal generation of the two techniques hints at the possibility of such studies also being feasible using AFM-IR.

### Image Processing and Data Analysis

Thermal drift is a known and common problem affecting AFM-IR measurements, particularly when these are conducted over longer periods of time, resulting in unintentional position changes. Especially when calculating ratios of two AFM-IR maps acquired at different wavenumbers, image alignment is crucial to avoid artifacts at the edges of features. Thermal drift correction is carried out by phase cross-correlation using the topography maps, which are recorded at the same time as the AFM-IR maps. However, the absence of a community-wide consensus on how to implement this drift-correction approach has led to widely different software implementations being employed, including Anasys Studio,^76^ SPIP,^53,117^ Python,^127^ and Matlab.^60^ Furthermore, beyond this simple step of data (pre-) processing, little discussion is happening within the community regarding data evaluation, i.e., how multivariate methods like PLSs should be applied to AFM-IR data sets, which types of normalization, smoothing and “bad pixel” removal are acceptable in AFM-IR data sets to retrieve reproductible spectra and images, and so forth. We expect that as the number of groups working on AFM-IR increases, especially those with a focus on the life sciences that most require multivariate methods for evaluation, this aspect will move toward the center of attention.

## Conclusion

In the last five years, AFM-IR has been applied to increasingly complex biological samples, from single DNA molecules^55^ and proteins^117^ to breast cancer tissue sections.^88^ The technique is increasingly moving from proof-of-concept studies toward application to practical problems where its potentially nondestructive nature allows for integration with other established microscopy techniques to obtain complementary information. We have summarized some of the recent applications of AFM-IR to the life sciences, identifying potential future directions of research, as well as relevant current limitations. AFM-IR’s high lateral resolution allows for an unprecedented access to chemical information on whole cells, cell components, and biodeterioration of materials. We hope that this perspective encourages more interdisciplinary collaboration between the life sciences and the growing field of AFM-IR by highlighting its potential contributions to challenges relevant to the life sciences.

## Data Availability

Experimental details of the data in Figure 4 are available upon reasonable request to the corresponding author.
